# Stage III Esophageal Squamous Cell Carcinoma Patients With Three-Dimensional Conformal or Intensity-Modulated Radiotherapy: A Multicenter Retrospective Study

**DOI:** 10.3389/fonc.2020.580450

**Published:** 2020-10-06

**Authors:** Qin Qin, Xiaolin Ge, Xin Wang, Lan Wang, Chen Li, Junqiang Chen, Xiaomin Wang, Yidian Zhao, Wencheng Zhang, Ping Wang, Qingsong Pang, Kaixian Zhang, Ling Li, Miaomiao Hu, Chongli Hao, Gaofeng Li, Yonggang Xu, Xueying Qiao, Zhiguo Zhou, Shuchai Zhu, Wenbin Shen, Yati Wang, Na Lu, Miaoling Liu, Shuai Qie, Chun Han, Zefen Xiao, Xinchen Sun

**Affiliations:** ^1^Department of Radiation Oncology, The First Affiliated Hospital of Nanjing Medical University, Nanjing, China; ^2^Department of Radiation Oncology, National Cancer Center/National Clinical Research Center for Cancer/Cancer Hospital, Chinese Academy of Medical Sciences and Peking Union Medical College, Beijing, China; ^3^Department of Radiation Oncology, The Fourth Hospital of Hebei Medical University, Shijiazhuang, China; ^4^Department of Radiation Oncology, Fujian Cancer Hospital/Fujian Medical University Cancer Hospital, Fuzhou, China; ^5^Department 4th of Radiation Oncology, Anyang Cancer Hospital, Anyang, China; ^6^Department of Radiation Oncology, Tianjin Medical University Cancer Institute and Hospital/National Clinical Research Center for Cancer, Tianjin, China; ^7^Department of Oncology, Tengzhou Central People's Hospital, Tengzhou, China; ^8^Department of Radiation Oncology, National Center of Gerontology, Beijing Hospital, Beijing, China; ^9^Department of Radiation Oncology, The Seventh Medical Center of People's Liberation Army of China General Hospital, Beijing, China; ^10^Department of Radiation Oncology, Affiliated Hospital of Hebei University, Baoding, China

**Keywords:** esophageal squamous cell carcinoma, three dimensional conformal radiotherapy, intensity-modulated radiotherapy, prognostic factor, survival

## Abstract

**Aim:** To evaluate long-term outcome and prognostic factors of stage III esophageal cancer after definitive radiotherapy using three dimensional conformal radiotherapy (3DCRT) or intensity-modulated radiotherapy (IMRT) techniques.

**Methods:** Patients with T3N1M0/T4N0-1M0 esophageal squamous cell carcinoma (ESCC) treated with definitive radiotherapy from 2002 to 2016 in 10 Chinese medical centers were retrospectively analyzed. Overall survival (OS) and progression-free survival (PFS) rates were calculated. Prognostic factors were analyzed by Log-rank test and multivariable Cox model.

**Results:** Survival data of 1,450 patients were retrospectively collected. With a median follow-up time of 65.9 months, 1-, 3-, and 5-year OS rates were 69.3, 36.7, and 27.7%, respectively, and PFS rates were 58.6, 32.7, and 27.4%, respectively. Univariable analyses showed that gender, age, lesion location, lesion length, largest tumor diameter, lymph node metastasis, gross tumor volume, EQD2, short-term response, and concurrent chemotherapy were prognostic factors for OS. Multivariable analyses showed that lesion location, T-classification, GTV size, EQD2, and short-term response to RT were independent prognostic factors for OS, and tumor diameter, GTV size, and short-term response were independent prognostic factors for PFS.

**Conclusions:** This study demonstrated that definitive radiotherapy using 3DCRT and IMRT provides promising outcomes for locally advanced ESCC.

## Introduction

Esophageal squamous cell carcinoma (ESCC), a major histopathologic subtype of esophageal cancer, is an aggressive disease with more than 50% of patients diagnosed at the unresectable stage (1). Definitive chemoradiotherapy (CRT) is recommended as a standard regimen for patients with locally advanced, unresectable esophageal cancer (2, 3). Two-dimensional (2D) conventional radiotherapy was the standard treatment for ESCC with a 5-year survival rate of only 10% (4–6). Intraluminal brachytherapy is recommended for patients unable to tolerate definitive CRT or to alleviate dysphagia in people with advanced esophageal cancer (7). The latest NCCN guideline recommends brachytherapy as an alternative to external-beam radiotherapy (EBRT) when dealing with complete esophageal obstruction if a lumen can be restored that allows for the use of appropriate applicators (8). Italian Association of Radiotherapy and Clinical Oncology (AIRO) has conducted a systematic review to examine efficacy of brachytherapy in esophageal cancer compared with other treatments and found that brachytherapy group had a median dysphagia-free survival (DyFS) of 99 days (9). In addition to palliative aim, brachytherapy can be also used for boost after EBRT or reirradiation. Because it offers a remarkable dose gradient allowing best organ at risk sparing, with an encouraging rate of long survivors (10). The advent of three-dimensional conformal radiotherapy (3DCRT) and intensity-modulated radiotherapy (IMRT) has provided advantages by improving dose conformity and reducing the radiation exposure of normal tissues. The advanced radiotherapy technologies have improved the 5-year survival to 20–30% (4–6). However, high levels of evidence to support their usage are scarce. Therefore, a large-scale retrospective study is urgently needed to evaluate the usefulness of 3DCRT or IMRT for ESCC. The current multicenter study conducted by 3JECROG aims to retrospectively report long-term outcome and prognostic factors of definitive 3DCRT/IMRT alone or in combination with chemotherapy in locally advanced ESCC of stage III.

## Materials and Methods

### Study Cohort

This work was approved by the institutional review board in accordance with the Declaration of Helsinki. Informed consent was obtained from each participant. Medical records of patients with ESCC treated between 2002 and 2016 with definitive radiotherapy (RT) in 10 Chinese medical centers were retrospectively analyzed. The eligibility criteria were as follows: age ≥ 18, clinically staged as T3N1M0/T4N0-1M0 (stage III) according to AJCC 6th edition, treated by definitive 3DCRT/IMRT with or without chemotherapy, received no previous treatment, histopathologically confirmed squamous cell carcinoma located in the esophagus or esophagogastric junction, Karnofsky performance score ≥ 70, available clinicopathological data, and follow-up period of no < 3 months in living patients. Patients who had undergone primary tumor resection or prior CRT were excluded.

### Treatment

All included patients had undergone RT delivered by 3DCRT or IMRT techniques. The plan was designed using Varian Eclipse or Elekta Monaco treatment planning system (TPS) with 6 MV photon beams from Varian Clinac or Elekta Precise accelerator, respectively. The IMRT plan had 5, 7, or 9 fields coplanar radiated fields. A forward-optimized 4–8 coplanar fields plan was designed. The beam number, directions, and the weights were manually adjusted. The gross tumor volume (GTV) was defined as any visible primary tumor (GTVp) plus metastatic lymph nodes (GTVnd) detected by CT, esophagogram, and endoscopy. The clinical target volume (CTV) included a 0.8–1.0 cm margin on either side of the GTVp or GTVnd, and 3.0–5.0 cm margin at long axis of GTVp. The planning target volume (PTV) was obtained by adding a 0.5 cm margin around the CTV. PGTV was determined by GTV plus 0.5 cm margin. The sequential boost or simultaneous integrated boost approaches were applied with a prescribed dose of 40.0–74.4 Gy to PGTV in standard 1.8–2.0 Gy fraction, 5 fractions per week. Isodose line of 95% prescription dose included 100% PTV and the volume receiving 104.5% of the prescription was limited to 5%. Dose–volume histograms (DVHs) were used to confirm that radiation plans optimized target coverage as well as normal tissue sparing. Planning objective for organs at risk (OARs) were defined as follows: total lung receiving more than 20 Gy (V20) ≤ 30%; maximum dose (Dmax) of spinal cord ≤ 50 Gy; for heart, with constraints: D1/3 ≤ 50 Gy, D2/3 ≤ 45 Gy, D3/3 ≤ 40 Gy (Dx means dose received by x of the volume). Homogeneity index (HI) of PTV calculated with HI = (D_2%_-D_98%_)/prescription dose 100%. Concurrent chemotherapeutic regimens were platin-based, including 5-FU-cisplatin, paclitaxel-cisplatin, and oxaliplatin-capecitabine. During the treatment, forty of the patients were re-evaluated operable after 40Gy radiotherapy and went on to surgery.

### Toxicity Evaluation and Follow-Up

Patients were clinically evaluated weekly during treatment, and laboratory parameters were examined. The first follow-up visit was performed 4–6 weeks after radiotherapy. Afterward, follow-up was conducted every 3 months for the first 2 years, every 6 months for the 3rd year, and annually thereafter. The follow-up evaluation included physical examination, blood testing, chest CT, esophagogram, and abdominal sonography. Short-term response was first evaluated on the completion date of RT and was reassessed after 1–3 months according to Response Evaluation Criteria for Solid Tumors version 1.1. Radiation-induced reactions were evaluated including pneumonitis, esophagitis, neutropenic fever, nausea/vomiting, anorexia, fatigue, weight loss, etc. Toxicities were evaluated using the Common Terminology Criteria for Adverse Events Version 3.0 (11).

### Statistical Analysis

The overall survival (OS) was defined as the time from the start of RT to death or the last follow-up. The progression-free survival (PFS) was calculated from the start of RT to disease progression, death, or the last follow-up. The OS and PFS were calculated using the Kaplan-Meier method. Differences between survival curves were analyzed using the log-rank test, and potential univariable prognostic factors were identified. The Cox proportional hazard model was used for multivariable analysis by incorporating significant univariable factors. The observed difference was considered statistically significant if the *P* < 0.05. All statistical analyses were conducted using SPSS version 21.0 (SPSS Inc., Chicago, IL, USA).

## Results

### Patients' Characteristics

A total of 1,450 patients were enrolled in this study according to the inclusion criteria and all patients have finished their treatment. Among them, 1,006 patients (69%) were male, and the median age was 65 years (30–90 years). The median follow-up period was 65.9 months (1.5–141.2 months). The total median dose delivered was 6,000 cGy. A total of 620 patients underwent concurrent chemotherapy and 239 received adjuvant chemotherapy. The clinicopathological characteristics of the patients are summarized in Table 1.

**Table 1:** Univariate analysis of overall survival (OS).

	**No. of patients**	**OS rate (%)**			**Median survival time (months)**	***P*-value**
		**1-year**	**3-year**	**5-year**		
**Age (years)**						
< 70	932	69.9	39.0	30.6	22.2	0.011
≥70	518	68.3	32.8	22.7	20.9	
**Gender**						
Male	1,006	66.9	34.7	25.8	20.6	0.003
Female	444	74.9	41.2	31.8	26.5	
**Lesion location**						
Cervical and upper-thoracic	489	74.6	43.3	33.0	25.6	<0.001
Middle-thoracic	660	66.2	31.9	23.6	19.5	
Lower-thoracic and esophagogastric junction	179	69.8	35.3	26.2	21.7	
**Length of lesion (cm)**						
≤ 5	730	73.4	39.6	31.6	23.9	0.001
> 5	720	65.1	33.7	23.6	19.2	
**The largest diameter of tumor (cm)**						
≤ 4	784	72.7	40.3	30.8	24.3	<0.001
> 4	409	62.5	32.4	23.3	17.1	
**Metastasis of lymph node**						
Yes	1,161	68.8	34.4	26.6	20.9	0.038
No	289	72.0	45.6	31.8	27.1	
**T stage**						
T3	511	72.7	36.5	30.6	23.9	0.063
T4	938	67.5	36.7	26.1	20.4	
**Radiation technique**						
3DCRT	493	70.3	38.2	28.1	22.6	0.704
IMRT	955	68.8	35.8	27.5	21.4	
**GTVp+GTVnd (cm**^**3**^**)**						
≤ 53	800	76.1	43.2	33.7	27.4	<0.001
>53	650	60.9	28.6	20.1	16.7	
**RT dose (EQD2, Gy)**						
40–50.4	70	56.4	23.4	14.0	16.0	0.029
>50.4	1,380	69.8	37.1	28.0	22.2	
**RT protocol**						
SIB-RT	380	68.8	37.3	28.1	21.9	0.892
SB-RT	134	65.9	34.6	28.9	19.2	
NB-RT	936	70.1	36.9	27.5	22.4	
**Concurrent chemotherapy**						
Yes	620	71.8	41.0	32.5	23.5	0.004
No	791	67.3	33.8	24.5	20.0	
**Adjuvant chemotherapy**						
Yes	239	71.8	39.7	33.2	21.6	0.135
No	1,172	68.5	36.2	26.7	21.5	
**Short-term response**						
CR	274	77.3	46.4	37.3	31.0	<0.001
PR+NR	528	65.5	32.9	23.8	18.4	
**Radiation induced esophagitis**						
Grade 0-1	980	70.0	36.8	27.1	22.2	0.672
Grade ≥2	306	69.7	38.5	29.5	23.1	
**Radiation induced pneumonitis**						
Grade 0-1	942	69.9	38.6	28.9	22.4	0.418
Grade ≥2	66	66.7	33.8	25.1	21.5	

*SIB, simultaneously integrated boost; SB, sequential boost; NB, no boost; CR, complete response; PR, partial response; NR, no response*.

### Survival and Safety Profile

**Figure 1:**
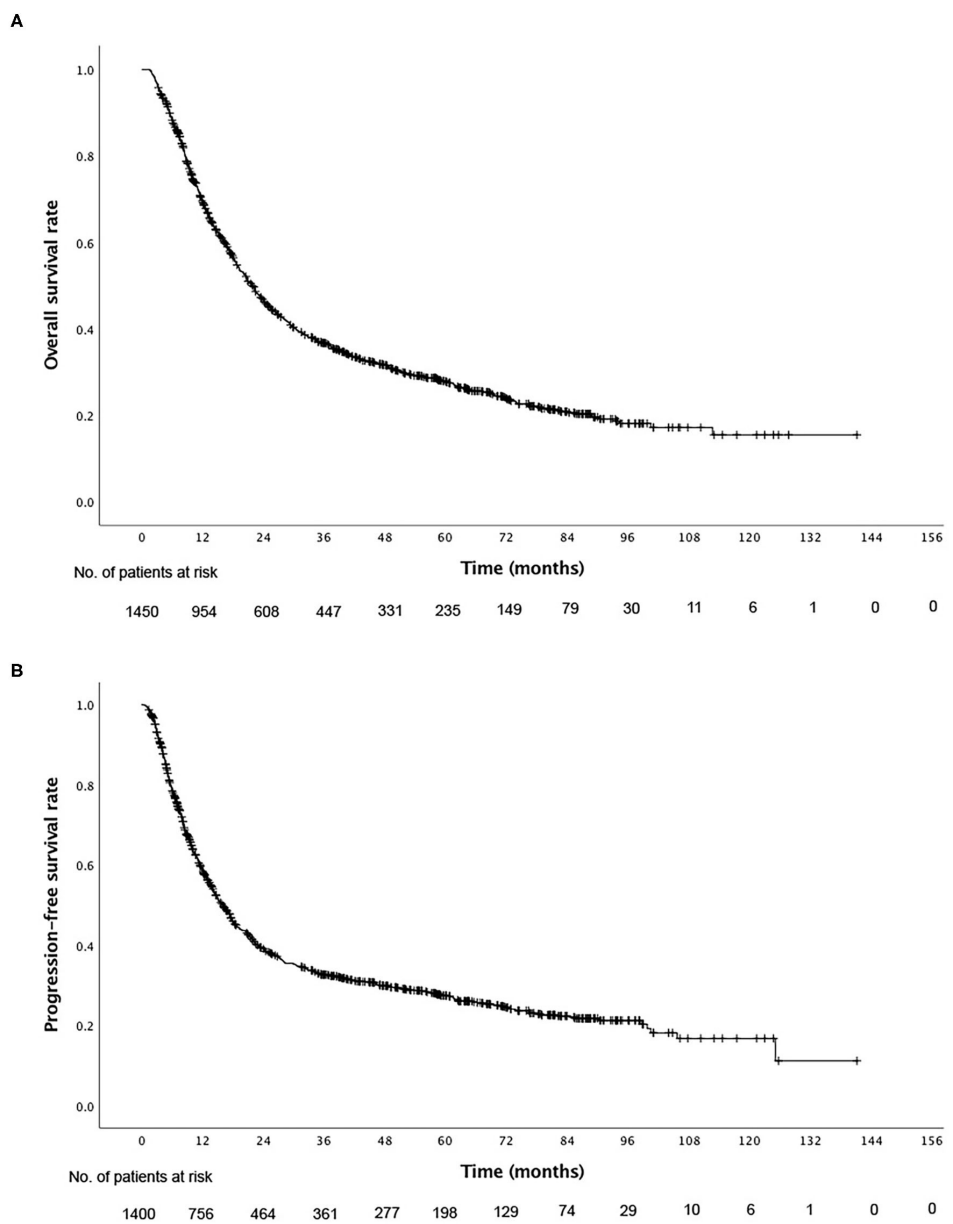
Kaplan–Meier curves of **(A)** overall survival and **(B)** progression-free survival for all patients.

For the total cohort, the 1-, 3-, and 5-year overall survival (OS) rates were 69.3, 36.7, and 27.7% (Figure 1A), and the PFS rates were 58.6, 32.7, and 27.4%, respectively (Figure 1B). Among the 1,016 patients who died, 792 (78.0%) patients died of esophageal cancer, 122 (12.0%) died of comorbidities, 24 (2.4%) died of complications following treatment (including acute radiation pneumonitis, esophagotracheal fistula, pulmonary infection, and radiation esophageal stenosis), 7 (0.7%) died from accident, and 68 (6.7%) died from unknown cause during the follow-up period. The rates of ≥Grade 2 acute esophagitis and pneumonitis were 306 (23.8%) and 66 (6.5%), respectively. 107 (7.9%) patients developed ≥Grade 2 weight loss. The incidence of neutropenic fever was 128 (9.6%). Apart from these adverse events, the treatments were well-tolerated in all patients. No treatment related nausea/vomiting/fatigue of grade 3 or higher occurred.

### Univariable Analyses

Results of univariable analyses for possible prognostic factors are summarized in Tables 1, 2. Patients who were male, ≥ 70 years old, had tumor in the lower portion of the esophagus, length of lesion > 5 cm, the largest diameter of tumor > 4 cm, metastasis of lymph node, GTV >53 cm^3^, EQD2 ≤ 50.4 Gy, non-complete response, and received no concurrent chemotherapy had worse OS than patients without these factors (Table 1). In contrast, male, distal tumor location, length of lesion > 5 cm, the largest diameter of tumor > 4 cm, lymph node metastasis, poor short-term response to radiotherapy, and large GTV were factors associated with worse PFS (Table 2).

**Table 2:** Univariate analysis of progression-free survival (PFS).

	**No. of patients**	**PFS rate (%)**			**Median survival time (months)**	***P*-value**
		**1-year**	**3-year**	**5-year**		
**Age (years)**						
< 70	902	59.3	34.1	29.1	16.4	0.153
≥70	498	57.3	30.4	24.5	15.4	
**Gender**						
Male	965	55.9	30.4	24.9	14.4	0.001
Female	435	64.5	37.6	32.8	20.4	
**Lesion location**						
Cervical and upper-thoracic	474	63.3	38.6	33.3	18.5	0.002
Middle-thoracic	634	56.6	28.2	23.0	14.8	
Lower-thoracic and esophagogastric junction	175	59.3	33.2	27.6	17.8	
**Length of lesion (cm)**						
≤ 5	718	61.2	35.6	30.1	17.4	0.018
> 5	682	55.9	29.5	24.6	14.3	
**The largest diameter of tumor (cm)**						
≤ 4	762	61.8	36.0	30.6	17.8	0.002
> 4	383	52.8	27.7	22.2	13.0	
**Metastasis of lymph node**						
Yes	1,123	57.5	31.0	25.5	15.0	0.013
No	277	63.1	39.4	35.1	18.2	
**T stage**						
T3	499	60.7	35.2	28.8	17.4	0.232
T4	900	57.4	31.4	26.7	15.2	
**Radiation technique**						
3DCRT	485	62.9	31.5	27.1	17.4	0.664
IMRT	913	56.2	33.7	27.4	14.8	
**GTVp+GTVnd (cm**^**3**^**)**						
≤ 53	800	64.8	37.2	30.7	19.5	<0.001
>53	600	50.2	26.6	23.0	12.2	
**RT dose (EQD2, Gy)**						
40–50.4	65	50.5	31.9	25.5	12.2	0.322
>50.4	1,335	58.9	32.8	27.6	16.1	
**RT protocol**						
SIB-RT	368	50.8	32.7	27.2	12.4	0.268
SB-RT	132	56.2	32.2	31.1	14.4	
NB-RT	900	62.0	33.1	27.2	17.1	
**Concurrent chemotherapy**						
Yes	604	57.4	35.4	30.7	15.9	0.479
No	757	60.5	31.2	25.5	16.6	
**Adjuvant chemotherapy**						
Yes	236	55.0	32.1	27.5	14.2	0.587
No	1,125	60.0	33.0	27.5	17.0	
**Short-term response**						
CR	268	65.5	38.9	34.8	22.9	0.001
PR+NR	510	59.3	28.1	22.3	15.4	
**Radiation induced esophagitis**						
Grade 0-1	949	58.3	32.3	26.9	15.9	0.767
Grade ≥2	292	57.6	33.0	28.4	16.0	
**Radiation induced pneumonitis**						
Grade 0-1	906	61.3	33.4	27.8	17.3	0.954
Grade ≥2	61	56.2	31.9	29.9	17.8	

*SIB, simultaneously integrated boost; SB, sequential boost; NB, no boost; CR, complete response; PR, partial response; NR, no response*.

### Multivariable Analyses

Multivariable analyses were performed using a Cox proportional hazards model, including possible prognostic factors. The results of multivariable analysis for OS revealed low tumor location, late T classification, large GTV, low dose of radiation, and poor short-term response to RT as independent prognostic factors associated with worse OS. In multivariable analysis for PFS, the diameter of tumor, size of GTV, and short-term response were significant prognostic factors (Table 3).

**Table 3:** Multivariate analysis of prognostic factors related to OS and PFS after 3DCRT/IMRT.

**Endpoint**	**Variable**	**HR**	**95% CI**	***P*-value**
OS	Lesion location			
	Middle-thoracic *vs*. Cervical/upper-thoracic	1.46	1.20–1.78	<0.001
	T stage	1.23	1.01–1.49	0.038
	GTVp+GTVnd	1.36	1.14–1.62	0.001
	RT dose (EQD2)	0.49	0.25–0.95	0.034
	Short-term response	1.44	1.20–1.72	<0.001
PFS	The largest diameter of tumor	1.31	1.08–1.58	0.007
	GTVp+GTVnd	1.25	1.03–1.51	0.023
	Short-term response	1.41	1.18–1.70	<0.001

### Dosimetric Results

Dosimetric characteristics of target and organs at risk for IMRT and 3DCRT were summarized in Table 4.

**Table 4:** Summary of dosimetric results for IMRT and 3DCRT.

	**Parameters**	**IMRT**	**3DCRT**
PTV	Homogeneity index	0.10 ± 0.01	0.16 ± 0.03
Lung	V5 (%)	51.09 ± 20.29	54.58 ± 21.92
	V20 (%)	21.06 ± 7.73	22.16 ± 7.92
	V30 (%)	11.62 ± 5.77	11.87 ± 6.50
	Mean lung dose (Gy)	12.39 ± 4.72	12.64 ± 3.53
Heart	V30 (%)	21.15 ± 18.14	30.30 ± 24.31
	V40 (%)	15.91 ± 15.18	23.02 ± 23.03
Spinal cord	Maximum dose (Gy)	40.85 ± 6.59	41.68 ± 7.98

## Discussion

Radiotherapy has been a critical treatment option for patients with locally advanced esophageal cancer mainly comprising stage III cancer. However, the one-, three-, and 5-year overall OS rates with conventional two-dimensional radiotherapy (2DRT) are poor, ranging from 38.1–58.2%, 13.1–22.4%, and 8.4–15.5%, respectively (4–6). Compared with 2DRT, 3DCRT improves the homogeneity of dose distribution in tumor target volume and delivers the ideal doses to the tumor and surrounding normal tissues. 3D computed tomography-based radiotherapy could deliver 60 Gy prescription dose to a target volume as large as 100% GTV and 95% CTV (12). Although the advancement of 3DCRT has been confirmed in dosimetry studies, little is known about the importance of technical improvements to clinical outcomes. Herein, a large population-based retrospective study was conducted to report the survival benefits of 1,450 patients with stage III esophageal cancer.

In our cohort, the 1-, 3-, and 5-year OS rates were 69.3, 36.7, and 27.7%, respectively, lower than the data recently reported by Chen et al. in a randomized, multicenter clinical trial of 436 locally advanced esophageal squamous cell cancer(78, 54, and 43%) (13). The discrepancy might be explained by the younger age of enrolled patients (81.3% < 70 years) and total use of concurrent chemotherapy in Chen et al.'s trial. However, our OS results were remarkably higher than those of stage III N(+) ESCC patients reported by Zhang et al. with 1-, 3-, and 5-year OS rates of 55.1, 24.4, and 17.1%, respectively (14). Reasons may include the differences in staging methods and T stage distribution. In univariable analysis, female gender and young age might predict better survival. Consistently, Deng et al. reported that age was an independent prognostic factor of survival (15). However, Jiang et al. (16) and Wang et al. (17) did not find gender/age as significant prognostic factors in patients who had undergone 3DCRT. Their cohorts were assumed to be extremely small to detect significant difference (*n* = 130/132). Fan et al. (18) reported that in locally advanced ESCC patients who received 3DCRT alone, those with tumors in the upper esophagus had a high survival benefit. Wang et al. (17) also identified tumor site as a prognostic factor for T4 esophageal carcinoma treated with 3DCRT. Our data in this study confirm these findings. The median OS rates of cervical/upper, middle, and lower thoracic/esophagogastric junction were 25.6, 19.5, and 21.7 months, respectively.

In accordance with many retrospective studies (19), tumor size characterized by length or diameter of lesion was found as a positive prognostic factor affecting DFS and OS in our study. Gross tumor volume which is closely related to tumor size has also been confirmed to be a significant prognostic factor for ESCC. In a retrospective analysis of 187 ESCC patients treated with definitive radiotherapy, patients with GTV >39.41 cm^3^ had significantly poorer PFS and OS than those with GTV ≤39.41 cm^3^ (20). Chen et al. also reported that the total target volume (gross tumor volume of primary lesion and metastases lymph node) were prognostic for overall survival (19). Owing to the relatively late stage, the median value of total GTV in the cohort was 48.7 cm^3^, larger than that of other studies. In our univariable analysis, an optimum cut-off point of GTV 53 cm^3^ was set for survival prediction and multivariable analysis, which identified GTV > 53 cm^3^ as an independent prognostic factor for poor PFS and OS, and confirmed the prognostic importance of GTV. A large GTV indicates heavy tumor burden, containing increased number of radioresistant hypoxic cells, and leading to decreased loco-regional control and worse survival.

Although the RTOG 94-05 trial identified no OS benefit from a high dose of 64.8 Gy compared with standard dose of 50.4 Gy (21), radiation dose escalation for esophageal carcinoma remains an ongoing debate. Several large population-based retrospective studies showed the survival benefit of having a dose ≥60 Gy. Chang et al. investigated the outcome of 2,061 patients with thoracic ESCC receiving IMRT-based concurrent CRT and observed that high radiotherapy dose (≥60 Gy) yielded favorable survival outcome in patients with advanced-stage (IIIA-IIIC) ESCC (22). Kim et al. showed similar results (23), indicating that a high dose (> 60 Gy) was associated with improved PFS and OS in patients with stage II–III esophageal carcinoma underwent definitive CRT. However, the analyses based on data from the National Cancer Database (24) failed to observe any long-term outcome benefit from dose escalation. This may be due to the inclusion of a limited number of patients who underwent IMRT or 3DCRT (39%), and the high proportion of unknown RT modality (61%) could have attenuated positive effect of RT escalation on survival by modern RT techniques. Similarly, the increased radiation toxicity due to dose escalation of 2D-RT partially contributed to non-significant result of RTOG 94-05. The development of 3D-CRT and IMRT enables the escalation of radiation dose with small fields, thereby extremely reducing radiation toxicity to normal tissues and highlighting the survival benefit of using a high dose. Moreover, an increasing number of studies using 3D-CRT or IMRT technique demonstrated the survival benefit of high-dose radiation. A recent retrospective study reviewed 115 consecutive EC patients treated with concurrent CRT and reported that patients who received higher dose RT (≥66 Gy) had better survival outcome than those given low dose RT (<66 Gy) with acceptable RT-related toxicities (25). Furthermore, dose escalation may be beneficial to late-stage disease. The NCCN guideline recommends 50–50.4 Gy as a standard radiation dose for locally advanced EC (26). In this study, we found that patients receiving radiation a dose of >50.4 Gy had a 5-year survival rate of 28%, which was twice that of ≤50.4 Gy (14%). However, a lot of patients in our cohort did not received concurrent chemotherapy. Hence higher RT dose could have compensated for lack of chemotherapy. To exclude this factor, we additionally compared the survival difference between higher and lower RT dose only in those receiving concurrent chemoradiotherapy. We found that the high-dose (≥60 Gy) group had significantly higher median PFS than low-dose (<60 Gy) group (16.40 vs. 11.13%; *P* = 0.028), while not significantly improved median OS (24.87 vs. 18.97%, *P* = 0.244). These findings suggest that high radiation dose on tumor by modern RT techniques results in better long-term outcome in patients with stage III ESCC.

Furthermore, this study showed that patients who achieved CR after radiotherapy had better overall survival and PFS rates than those who achieved non-CR. Moreover, the short-term response was identified as an independent prognostic factor of OS and PFS. A prospective randomized RTOG 85-01 study established concurrent CRT as the standard care for unresectable esophageal cancer (3). Consistently, the results of the long-term follow-up study suggested that concurrent CRT yielded a survival benefit for locally advanced ESCC compared with radiation treatment alone. Up to now, few studies examine the efficacy of adjuvant chemotherapy following RT in patients with EC. Neither a Japanese randomized clinical trial nor Chinese retrospective study has demonstrated evident survival benefits of adjuvant chemotherapy compared with RT/CRT-alone (27, 28). Data from the present multi-center study support the conclusion reported in previous studies.

Although 3DCRT/IMRT is the standard RT technique for EC, as used in the current study, 306 and 66 patients, respectively, suffered from severe radiation induced esophagitis and pneumonitis and 24 patients died from treatment related complications. Thus, newer technologies such as proton beam therapy (PBT) that exploits physical properties inherent to heavier particles have gained extensive attention to reduce radiation dose exposure to nearby organs at risk. Numerous dosimetric studies have illustrated superior cardiopulmonary dose sparing with PBT compared with both 3DCRT and IMRT (29–32). A newly reported randomized phase IIB trial compared total toxicity burden (TTB) and PFS between PBT and IMRT for esophageal cancer (33). The results suggested that PBT for neoadjuvant or definitive treatment of locally advanced EC produced a lower toxicity profile and fewer postoperative complications, thus leading to a lower TTB, but similar PFS, compared to IMRT. In addition to PBT, other novel RT techniques, e.g., volumetric-modulated arc therapy (VMAT), image guided radiation therapy (IGRT) and, Helical Tomotherapy are also promising in treatment of EC.

There are several limitations in the present study. First, the retrospective design might have introduced selection bias. Second, the multicenter data collection allowed to increase the sample size, but it also resulted in heterogenous cohort and variable treatment regimens. Moreover, not all patients were treated with standard of care (concurrent chemoradiotherapy). Thus, a large prospective study with rigorous criteria is warranted to confirm our findings.

In conclusion, the long-term outcome of 3DCRT/IMRT for ESCC is encouraging. Lesion location, T stage, GTV size, radiation dose, and short-term response are prognostic factors associated with OS for stage III ESCC patients treated with definitive RT. Our findings may provide additional prognostic information to the clinical decision making for ESCC.

## Data Availability Statement

All datasets presented in this study are included in the article/supplementary material.

## Ethics Statement

The studies involving human participants were reviewed and approved by Nanjing Medical University. The patients/participants provided their written informed consent to participate in this study.

## Author Contributions

XS designed the study. QQ, XG, XinW, LW, CL, JC, XiaW, YZ, WZ, PW, QP, KZ, LL, MH, CH, GL, YX, XQ, ZZ, SZ, WS, YW, NL, ML, SQ, CH, and ZX collected and analyzed the data. All authors contributed to the article and approved the submitted version.

## Conflict of Interest

The authors declare that the research was conducted in the absence of any commercial or financial relationships that could be construed as a potential conflict of interest.
